# Do All Portable Cases Constructed by Caddisfly Larvae Function in Defense?

**DOI:** 10.1673/031.013.0501

**Published:** 2013-01-24

**Authors:** Emily E. Ferry, Gareth R. Hopkins, Amber N. Stokes, Shabnam Mohammadi, Edmund D. Brodie, Brian G. Gall

**Affiliations:** 1 Department of Biology, Utah State University, 5305 Old Main HL, Logan UT 84322; 2 Ecology Center, Utah State University, 5305 Old Main HL, Logan UT 84322; 3 Department of Biology, Hanover College, PO Box 108, Hanover IN 47243

**Keywords:** dragonfly, Odonata, survival, Trichoptera

## Abstract

The portable cases constructed by caddisfly larvae have been assumed to act as a mechanical defense against predatory attacks. However, previous studies have compared the survival of caddisflies with different cases, thereby precluding an analysis of the survival benefits of “weaker” case materials. The level of protection offered by caddisfly cases constructed with rock, stick, or leaf material, as well as a no-case control, was investigated against predatory dragonfly nymphs (*Anax junius* Drury (Anisoptera: Aeshnidae)). A valid supposition is that the cases made of stronger material are more effective at deterring predators. Yet, observations revealed that there was no difference in survival between the case types. All caddisflies with a case experienced high survival in comparison to caddisflies removed from their case. In addition, larvae with stick-cases experienced fewer attacks and captures by dragonflies. These results showed that the presence of a case, regardless of the material used in its construction, offers survival benefits when faced with predatory dragonfly nymphs.

## Introduction

Examining an organism's predator-prey interactions often provides insight into the causation of their behaviors and the evolution of morphological characteristics (Lima and Dill 1990). Caddisflies (Trichoptera) possess a unique combination of traits that have facilitated their diversification in almost all freshwater ecosystems (Peckarsky 1982; Wiggins 2004). These aquatic larvae manufacture cases using different materials from the environment (Mackay and Wiggins 1979). These materials consist of organic particles, including pieces of leaves, sticks, or bark, as well as inorganic material such as sand (Wiggins 2004). The cases may be much larger and heavier than the larva itself (e.g., Otto 2000; Gall et al. 2011), which necessitates the allocation of substantial resources to their production and movement (Otto and Johansson 1995; Otto 2000).

Despite the obvious costs of building and carrying a portable home, several hypotheses exist to explain the potential benefits of case construction. For example, Milne (1938) suggested the case may facilitate respiration in an aquatic environment, and Williams et al. (1987) presented empirical evidence indicating that this is indeed a function of cases in some species. Many aquatic predators forage using visual cues, and it has also been suggested that the cases function to camouflage the larva inside (Nielsen 1942). However, the most commonly assumed function of case construction is that it physically protects the larva during a predatory attack. A number of studies have empirically examined the effect of case material on the survival probability of the associated larvae (Otto and Svensson 1980; Johansson 1991; Johansson and Johansson 1992; Johansson and Nilsson 1992; Nislow and Molles 1993). For example, Otto and Svensson (1980) found that cases made of mineral material withstood substantially greater crushing forces than cases made of leaf and bark material. In addition, caddisflies inhabiting mineral cases were more likely to survive predatory encounters compared to caddisflies in cases made of leaf material (Otto and Svensson 1980). These studies have compared differences between case types, yet few studies have attempted to elucidate the benefits of the general presence of a case on the survival of caddisfly larvae against potential predators.

In the present study, caddisflies with one of three different case types, as well as a no-case control, were exposed to predatory dragonflies to determine whether (1) cases made of different material differentially affect caddisfly survival, and (2) what role the general presence of a case has on caddisfly survival compared to the absence of a case. The use of such a control offers greater insight to the degree of protection offered by these cases and allows one to empirically address whether case-building behavior functions as an antipredator mechanism.

## Materials and Methods

### Animal Collection and Maintenance

**Figure 1.**
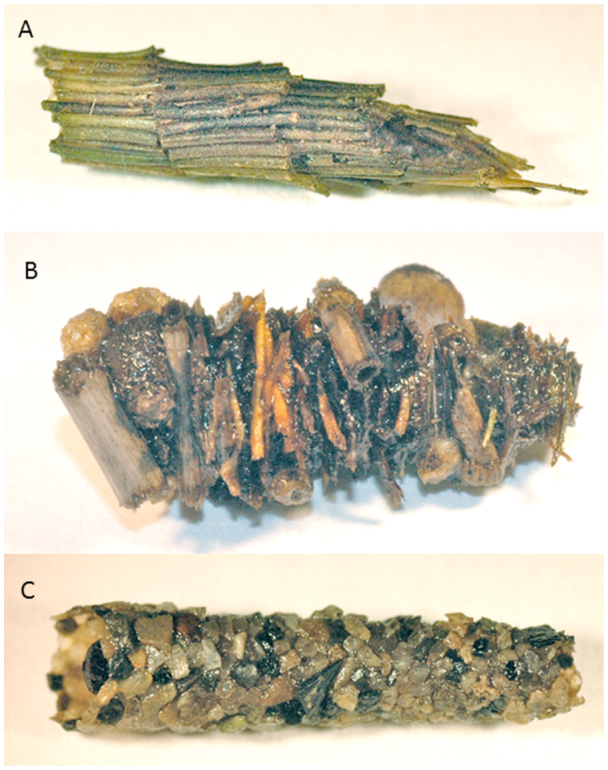
Three case types built by caddisfly larvae used in predation experiments. (A) “Leaf” case constructed by *Agrypnia deflata*. (B) “Stick” case built by *Limnephilus flavastellus*. (C) “Rock” case constructed by *Hesperophylax occidentalis*. High quality figures are available online.

Three species of caddisfly were used in this experiment, each constructing their case from a different material (Figure 1). *Agrypnia* sp. (likely *A. deflate* Milne (Trichoptera: Phryganeidae)) constructed cases of leaf material arranged in a spiral pattern (Figure 1A). The cases of *Limnephilus flavastellus* Banks (Trichoptera: Limnephilidae) were composed of stick and bark fragments arranged transversely (Figure 1B). *Hesperophylax* occidentalis Banks (Trichoptera: Limnephilidae) constructed cases of mineral material (Figure 1C). *L. flavastellus* (henceforth “stick-case”) were collected on 8 March 2011 from the Soap Creek ponds in Benton County, Oregon. *A. deflata* (henceforth “leaf-case”) were collected 24 August 2011 from a pond near Preston, Idaho. *H. occidentalis* (henceforth “rock-case”) were collected 26 September 2011 from a pond near Paradise, Utah. Both the leaf-case and stick-case caddisflies were maintained in 38-L aquaria with an aerator and maple leaf detritus after collection. They were kept in an environmental chamber at 6° C on a 12:12 L:D cycle. Twenty-four hours prior to testing, the stick-case and leaf-case caddisflies were transferred to 11-L plastic tubs, which were filled with 4 L of filtered tap water, detritus, and an aerator. These tubs were placed in an environmental chamber at 18° C. The detritus was prepared by placing dried maple leaves (*Acer*) into a container with filtered tap water, a small amount of pond water, and an aerator for several weeks prior to use to promote the buildup of beneficial bacteria and fungi. The rock-case caddisflies were transferred to the same 11-L plastic tubs and environmental chamber immediately after their collection.

Nymphs of the dragonfly *Anax junius* Drury (Odonata: Aeshnidae) were used as the predator for this study. Nymphs were collected from the same ponds as the leaf-case caddisflies, but due to their small size (mean total length ± SE = 18.17 ± 0.42) it is unlikely that the majority of nymphs collected were predators of caddisflies prior to experimentation; preliminary trials with nymphs not used in this experiment indicated they did not forage on caddisflies at this stage. Dragonfly nymphs were housed individually in round glass bowls (5 cm × 10 cm), with a small rock (approximately 2-cm diameter) for perching, and 225 mL of filtered tap water. These bowls were maintained in the 18° C environmental chamber. After collection, nymphs were maintained on a rigorous diet of blackworms, *Lumbriculus variegatus*, to stimulate development in order to attain a size suitable for experimentation. Food was withheld from dragonfly nymphs for 7 days prior to experimentation to stimulate feeding responses. The glass bowls were cleaned 5 days prior to experimentation.

### Experimental Protocol

Dragonfly larvae were offered caddisflies with one of four case-types, including caddisfly larvae removed from their case (N = 20), leafcase caddisflies (N = 21), stick-case caddisflies (N = 19), and rock-case caddisflies (N = 20). To begin a trial, an *A. junius* was randomly chosen and the bowl with the nymph was removed from the environmental chamber. A white blind was placed around the dish to minimize external visual influences and the *A. junius* was allowed to acclimate for 2 minutes. A treatment (no case, leaf, stick, or rock) was randomly selected. A caddisfly with the correct case type was randomly selected and removed with forceps from the appropriate tub. The length of its case was recorded; there was no difference in caddisfly case length among treatments (*p* = 0.08). The caddisfly was placed approximately two centimeters in front of the *A. junius*. As an antipredator defense, caddisflies remain inside their case following handling (Gall and Brodie 2009). The trial started when the caddisfly emerged from its case and started moving. For the no-case treatment, a species of caddisfly was randomly selected (all three species were equally represented) and the larva was gently removed from its case with a probe. It was then placed in front of the *A. junius*, at which time the trial began.

The following were recorded: the number of attacks, the number of captures, the time the *A. junius* spent holding the caddisfly, and whether the caddisfly was ingested or released. An attack was recorded whenever the *A. junius* nymph struck at the caddisfly with its labium. A capture was recorded when the *A. junius* attacked with its labium and seized the caddisfly. A trial ended after the caddisfly was ingested or after 10 minutes. At the conclusion of testing, the *A. junius* was removed from its dish and its length was recorded; there was no difference in *A. junius* length between treatments (*p* = 0.73). *A. junius* were never reused on the same day, but may have been reused once, 48 hours later.

To ensure that differences in survival were not due to differences in activity between the prey in each treatment, caddisfly activity was compared across the four treatments (no case (N = 15), all others (N = 7)). A caddisfly was placed in a round glass bowl (5 cm × 10 cm) with fiberglass mesh on the bottom. A fourquadrant grid was placed beneath the dish, and the number of lines crossed by each caddisfly was counted as it moved across the dish. The number of lines crossed was recorded during a five minute interval.

### Statistical Analyses

The effect of case type (no case, leaf, stick, or rock) on the number of attacks, number of captures, time spent grasping prey, and caddisfly activity (number of lines crossed) was assessed using a one-way ANOVA in a completely randomized design. Pairwise comparisons among the case types were adjusted for family-wise Type I error using the REGWQ method. The GLM procedure in SAS 9.1 (SAS Institute Inc.) was used for all calculations. Data were transformed to meet statistical assumptions where necessary. The proportion of caddisflies that survived was calculated for each treatment by dividing the number of individuals that were released and never attacked by *A. junius* nymphs by the total number of trials in that treatment. Excluding trials that did not yield an attack does not qualitatively change the results; including these data is likely a better representation of survival because these prey may possess a phenotype that is unacceptable or unpalatable to the predator. The survival of caddisflies in the four treatments was analyzed using a general linear mixed model with a binomial distribution and the logit link function in a completely randomized design. This analysis was followed by pairwise comparisons between case-type means using the Tukey-Kramer method. The mean and standard error for each treatment were then back transformed from the logit scale. This analysis was performed using the GLIMMIX procedure in SAS 9.2. To determine if the diameter of the caddisfly cases differed between case types, the diameter (at the widest point) of 10 cases from each species was measured with digital calipers. The difference in diameter between the three case types was compared using a one-way ANOVA followed by pairwise comparisons (REGWQ method).

**Figure 2.**
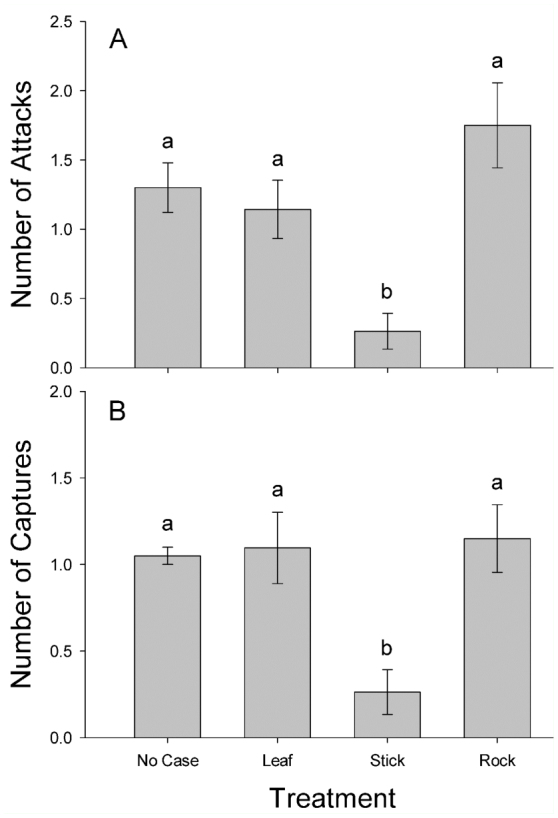
Mean (± SE) number of attacks (A) and number of captures (B) by *Anax junius* nymphs on caddisflies with one of four case types. Different letters indicate significant differences between treatments (*p* < 0.001). High quality figures are available online.

### Results

**Figure 3.**
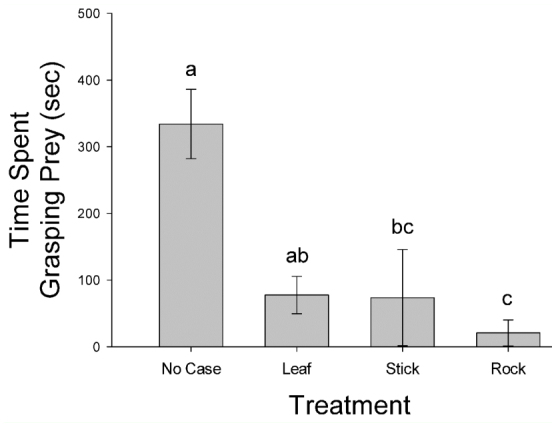
Mean (± SE) time *Anax junius* nymphs spent grasping caddisflies with one of four case types. Different letters indicate significant differences between treatments (*p* < 0.001). High quality figures are available online.

**Figure 4.**
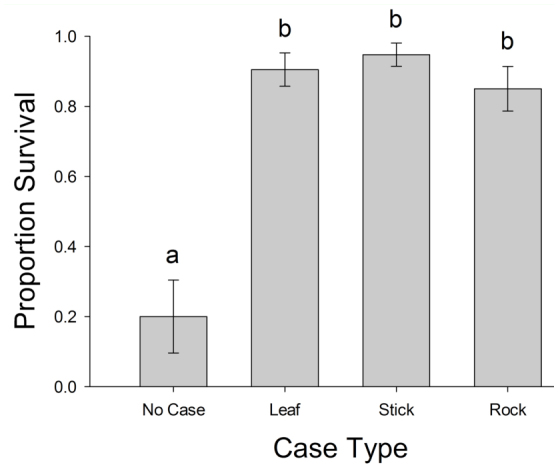
Proportion of caddisfly larvae with one of four case types that survived predatory encounters with *Anax junius* nymphs. Different letters indicate significant differences between treatments (*p* < 0.002). High quality figures are available online.

There was a significant difference in the number of attacks (F_[3,76]_ = 15.39, *p* < 0.0001; Figure 2A) and the number of captures (F_[3,76]_ = 13.08, *p* < 0.0001; Figure 2B) among the four case types. Caddisflies with stick cases received fewer attacks and fewer captures than caddisflies with rock, leaf, or no case (Figure 2). There was a significant difference between treatments in the time *A. junius* nymphs spent grasping prey (F_[3,36]_ = 20.14,*p* < 0.0001; Figure 3), with caddisflies removed from their case generally being grasped for a longer period of time than caddisflies with a case (Figure 3). There was a significant difference in survival between the case types (df = 3, χ^2^ = 36.14, *p* < 0.0001; Figure 4). Caddisflies that had a case, regardless of material, were more likely to survive a predation event than individuals removed from their case (Figure 4).

The activity level (lines crossed) of caddisflies was not significantly different among case types (F_[3,32]_ = 1.26, *p*= 0.31). There was a significant difference in case diameter among the three types of cases (F_[2,27]_ = 74.45, *p* < 0.0001). Stick cases (mean diameter ± SE = 7.49 ± 0.27) were wider than leaf (mean diameter = 3.64 ± 0.09) and rock cases (mean diameter = 4.32 ± 0.30).

### Discussion

Other studies have documented the protection that caddisfly cases offer from predators (Otto and Svensson 1980; Johansson 1991; Johansson and Johansson 1992). However, these studies did not control for the presence or absence of a case, and therefore did not determine the relative protective value of different case materials. This study investigated whether the presence of a case, of any material, offers protection against predators. Strong evidence was found that caddisfly cases operate as a defensive mechanism against potential predators. While there was no significant difference in the proportion of leaf, stick, and rock-cased caddisflies surviving a predation event, all caddisflies with a case survived considerably better than those without a case. Several previous studies have documented the general protective value of possessing a case compared to individuals that have been experimentally removed from their case. For example, Wissinger et al. (2004) demonstrated that individual caddisflies that possessed a case were less likely to succumb to cannibalism compared to their caseless counterparts. Furthermore, although several species of caddisflies that build cases of different material had different survivorship against predatory salamanders, each had higher survival when left in their case relative to individuals of that species that were removed (Wissinger et al. 2006). These results, in conjunction with the results presented here, indicate that case construction is an important adaptation for reducing predation. Furthermore, even cases constructed from materials generally assumed to provide less protection can provide a survival benefit to their occupant against some predators.

We hypothesized that those case materials that have been experimentally documented to resist greater forces (i.e., mineral cases) would provide greater resistance against predation and increase chances of survival, as has been demonstrated previously (Otto and Svensson 1980; Johansson 1991; Nislow and Molles 1993; Wissinger et al. 2006). Although the relative strength of the cases used in this study was not measured, these cases qualitatively appeared to reflect this gradient; rock cases were generally stronger than stick cases, which were stronger than leaf cases (BGG personal observation). Previous studies investigating the role of caddisfly cases as antipredator devices primarily used fish as predators (Otto and Svensson 1980; Johansson 1991). In the studies cited, caddisflies with a rock case typically survived better than caddisflies with leaf or stick cases. It may be surprising, then, that there was no difference observed in our study in the degree of protection based on the material a caddisfly uses to construct its case. In another study using dragonfly nymphs (*Aeshna juncea*) as predators, the number of attacks, captures, and ingestions did not differ between caddisfly larvae with leaf cases and stick cases (Johansson and Johansson 1992). However, Nislow and Molles (1993) found that caddisflies were more likely to survive attacks against dragonflies (*Oplonaeschna armata*) when their cases contained a higher proportion of mineral material, although the authors interpreted this result with caution because the proportion of mineral material was assigned visually. Fish and invertebrate predators use different techniques to capture the prey; when a fish feeds on a caddisfly, it is ingested whole (Johansson 1991). Once inside the mouth, the fish will crack the case and either digest the case along with the caddisfly or spit the case out (Johansson 1991). On the other hand, dragonflies make direct contact with the labium when attacking the caddisfly (Corbet 1999). The dragonfly must chew their way through the case in order to ingest the caddisfly (Johansson and Johansson 1992). In our study, dragonflies released the cased caddisflies almost immediately after capture, and all three case types were sufficient to protect caddisflies and increase their probability of surviving the predation event.

Caddisflies without a case were grasped for longer periods of time by dragonflies. This was due to the fact that these caddisflies were without a case to shield them and were ingested. In this study, several cased caddisflies were ingested, and in each instance the dragonfly required a substantial amount of time to consume the larvae. Johansson and Johansson (1992) found that dragonfly predators either consumed caddisflies by seizing the portion of the larva that was outside the case or by chewing through the case wall. Substantially greater handling times were required when the dragonflies chewed through the cases (Johansson and Johansson 1992). This additional time could provide caddisfly larvae with an opportunity to escape the predation event by abandoning the case before it is breached.

Although stick-case caddisflies had similar survival compared to the other case types, they were attacked and captured less frequently. This may be best explained by stick cases having a greater overall diameter and appearing too large to consume. Otto and Johansson (1995) found that caddisfly larvae were more susceptible to predation when stones attached laterally on all sides of the cases were removed. The lateral stones made by caddisfly larvae look too large for predatory fish to consume (Otto and Johansson 1995; Otto 2000). Moreover, wider cases have been found to be three times more resistant to cracking than longer cases with smaller diameters (Johansson 1991). A larger case may deter predators because consuming such a case would require the expenditure of substantially more time and energy than alternative prey. In addition, caddisflies with this case-type may experience additional benefits that went unmeasured in our study. For example, if attacked and captured less frequently, caddisflies with stick cases would possess more time for other fitness enhancing activities such as foraging or reproduction (Lima and Dill 1990). Future work should focus on the proximate mechanisms leading to reduced attack rate for these caddisflies, as well as the possible benefits of such a defense.

Caddisflies build cases that function as protective armor against predators out of a variety of materials in their environment. The results of our study demonstrate that the presence of any case, constructed from even relatively weak materials, provides protection from at least some predators. Moreover, possessing a case that is larger than others may provide additional benefits.
